# Effects of Omega-3-Rich Pork Lard on Serum Lipid Profile and Gut Microbiome in C57BL/6NJ Mice

**DOI:** 10.1155/2022/9269968

**Published:** 2022-11-22

**Authors:** Anantawat Koontanatechanon, Manoosak Wongphatcharachai, Nutthawan Nonthabenjawan, Pichaya Jariyahatthakij, Thanchanok Khorporn, Wanpeuch Parnsen, Benjawan Keattisin, Pattarin Leksrisompong, Pairat Srichana, Sattrachai Prasopdee, Sittiruk Roytrakul, Kusuma Sriyakul, Veerachai Thitapakorn, Kammal Kumar Pawa

**Affiliations:** ^1^Chulabhorn International College of Medicine, Thammasat University, Pathumthani, Thailand; ^2^Feed Technology Office, Charoen Pokphand Foods Public Company Limited (CPF), Bangkok, Thailand; ^3^CPF Food Research & Development Center, Charoen Pokphand Foods Public Company Limited (CPF), Bangkok, Thailand; ^4^National Center for Genetic Engineering and Biotechnology (BIOTEC), National Science and Technology for Development Agency (NSTDA), Pathumthani, Thailand

## Abstract

**Background and Aims:**

Hyperlipidemia is a risk factor for cardiovascular diseases. This study is aimed at investigating the effects of consuming omega-3-rich pork lard on the serum lipid profile and gut microbiome of the mice model.

**Methods and Results:**

We divided 23 C57BL/6NJ males (16-week-old) into 3 groups, and each group received either a control diet, a high-fat diet of coconut oil (coconut oil), or a high-fat diet of omega-3-rich pork lard (omega lard) for 28 days. Thereafter, fasting serum lipids and fecal microbiomes were analyzed. The serum cholesterol, triglyceride, and LDL levels of the omega lard-treated group were significantly reduced compared to the coconut oil-treated group (*P* < 0.05). However, the microbiome analysis revealed a significant increase in the abundance of *Lachnospiraceae* in the omega lard-treated group compared to the coconut oil-treated group (*P* < 0.05). Furthermore, Spearman's correlation analysis revealed that the increased serum lipid content was positively correlated with the abundance of *Bacteroidaceae* (*P* < 0.05) and negatively correlated with the abundance of *Lachnospiraceae* (*P* < 0.05).

**Conclusions:**

These findings suggested that omega-3-rich pork lard altered the serum lipid profile and gut microbiome in the mice model. *Practical Application*. The excellent protection offered by omega-3-rich pork lard against hyperlipidemia indicated that pork lard could be used as alternative cooking oil for health-conscious individuals. It could also be introduced as a functional ingredient for patients with hyperlipidemia.

## 1. Introduction

Reports suggest an association between dietary nutrition and health or immunity, especially an association between deteriorated health and the Western diet, which contains high dietary fats [1]. Owing to the high content of omega-6 fatty acids and low proportion of vegetables and fibers in the Western diet, the prevalence of obesity and other metabolic diseases, including cardiovascular disease (CVDs) and nonalcoholic fatty liver disease (NAFLD), has increased significantly in the past years [2]. However, replacing it with a beneficial fatty acid source can potentially ameliorate a few of the metabolic diseases [3].

Several studies evaluated the health benefits of omega-3 supplements, including the improvement of metabolic parameters and cardioprotective effects in an animal model [4]. Improvement in hepatic steatosis, oxidative stress, and systemic inflammation has also been reported in a high-fat diet- (HFD-) fed mice model supplemented with omega-3 [5]. Extending upon the animal model, clinical trials involving parenteral nutrition have determined a significant reduction in infection risk, length of stay in the intensive care unit, length of stay in the hospital, risk of sepsis, and mortality rate among adult hospitalized patients [6].

In addition to the health benefits reported from omega-3 supplementation trials, a double-blinded clinical trial involving the consumption of omega-3-enriched eggs and chicken demonstrated improved diastolic blood pressure in healthy normotensive adults [7]. This indicated that omega-3 could exert health benefits through the consumption of enriched foods.

Moreover, an omega-3 supplement has been reported to manipulate the gut microbiome, including an increase in the abundance of *Bacteroidetes*, *Verrucomicrobia*, *Akkermansia*, *Lactobacillus*, and *Bifidobacteria* and a decrease in the abundance of *Firmicutes* and *Proteobacteria* [8]. Interestingly, a study reported that supplementation with omega-3 fatty acids favored the growth of short-chain fatty acid- (SCFA-) producing bacteria, including *Lachnospiraceae*, in the gut microbiome of HFD-fed C57BL/6J mice [9]. This indicated that omega-3 can also alter the gut microbiome of mice.

In this study, we investigated the health benefits of omega-3-rich pork lard, which contains pork lard obtained from pigs fed with omega-3-fortified feed [10]. Consumption of omega-3 fatty acids provides several health benefits; for example, an increase in omega-3 intake improves the omega-3/omega-6 fatty acid ratio in the diet. Therefore, similar benefits were expected upon consumption of omega-3-rich pork meat [11]. The feed of omega-3-rich pork lard-producing pigs is usually fortified with tuna oil or microalgae to increase the omega-3 content, especially eicosapentaenoic acid (EPA) and docosahexaenoic acid (DHA) [12]. However, for an economical reason, flaxseeds have been used to fortify pig's feed to produce pork meat with high alpha-linolenic acid (ALA) content, an essential omega-3 fatty acid found in nuts and plants [13].

This study is aimed at investigating the effects of omega-3-rich pork lard on the serum lipid profile and gut microbiome of C57BL/6NJ mice. The findings of this study will improve the understanding of the effects of omega-3-enriched food ingredients on health and gut microbiome and will lay the foundation for further research on this theme.

## 2. Materials and Methods

### 2.1. Animals

The animals were approved by the National Laboratory Animal Center-Animal Care and Use Committee (NLAC-ACUC), National Laboratory Animal Center, Mahidol University, Thailand, under protocol number RA2019-50. A total of 23 16-week-old male C57BL/6NJcl mice were obtained from Nomura Siam International (Bangkok, Thailand), housed in individual cages with shredded paper strips as enrichment, and provided ad libitum food and water. The animal room was equipped with a positive pressure ventilation system, with room temperature set at 22.3°C, 30–70% relative humidity, and a 12 h light cycle.

All animals were subjected to a one-week quarantine and one-week acclimatization to adapt to the environment, followed by four weeks of the experimental period.

### 2.2. Diet Preparation

Following the quarantine and acclimatization period, mice were randomly divided into the following three dietary groups: mice fed on a commercial rodent diet (082 diet; Perfect Companion Group Co., Ltd., Bangkok, Thailand) (control) (*n* = 8), mice fed on a 40% fat diet prepared using coconut oil (coconut oil) (*n* = 7), and mice fed on a 40% fat diet prepared using omega-3-rich pork lard (omega lard) (*n* = 8). The control diet was a standard chow diet, whereas the coconut oil and omega lard diets were prepared in agar. The fatty acid composition of the oils used and the macronutrient energy distribution of each diet are shown in Tables 1 and 2, respectively. The coconut oil diet and omega lard diet were made using food grade ingredients, calculated for specific calorie distribution of protein, carbohydrate, and fat. The diets were then blended with water and mixed with agar to the final of 1% agar to form homogeneous semisolid diets as described previously [14]. Approximately 10 g of agar was added to each 1 kg of diet, contributing approximately 2 kcal from the total of 1,756 kcal/kg of each diet. The composition of coconut oil diet and omega lard diet and the energy content of each component are shown in Tables 3 and 4, respectively (Supplementary Table S10). Mice were fed for four weeks on respective diets, and their body weights and food intake were recorded daily throughout the experimental period.

### 2.3. Collection of Blood, Liver, Visceral Fat, and Feces

Mice were first exposed to isoflurane anesthesia and then sacrificed by cervical dislocation. Thereafter, blood samples were collected by cardiac puncture, incubated at room temperature for 10 min, and centrifuged at 2,000 × *g* for 10 min at 4°C. The sera were collected and stored at -80°C until further analysis.

Subsequently, the liver and visceral fat were collected and weighed. The liver tissues were then subjected to histopathological examination using H&E-stained tissue slides, which were prepared as described in [15]. Briefly, the liver tissues were fixed in 10% formaldehyde, followed by preparation using a tissue processor Leica ASP 300s (Leica, Germany). The slides were then subjected to hematoxylin–eosin staining using Leica ST5010 Autostainer XL (Leica, Germany), followed by macrovesicular steatosis scoring. The reference for the diagnostic terms and glossary used for the histopathological examination can be obtained from the International Harmonization of Nomenclature and Diagnostic Criteria (IHAND) developed by Societies of Toxicological Pathology from Europe (ESTP), Great Britain (BSTP), Japan (JSTP), and North America (STP). The lesion scores were based on a 5-level scale, ranging from normal (0) to minimal (+1), mild (+2), moderate (+3), and severe (+4). The scale was applied semiquantitatively in direct proportion to the macrovesicular steatosis [16].

Feces were collected in a clean 1.5 mL centrifuge tube, frozen in liquid nitrogen, and then stored at -80°C until further analysis.

### 2.4. Biochemical Analysis of the Serum

A Cobas C311 Biochemistry Analyzer (Roche®, Switzerland) was used to measure serum concentrations of glucose, total cholesterol, triglycerides, low-density lipoprotein (LDL), and high-density lipoprotein (HDL). Furthermore, blood urea nitrogen (BUN) and creatinine levels were measured to determine the effects of omega-3-rich pork lard on the kidneys and the activity of alanine aminotransferase (ALT), whereas the activity of aspartate aminotransferase (AST) was measured to evaluate the effects on the liver.

The protocol for the biochemical analysis of serum using the equipment Cobas C311 was described by Koontanatechanon et al. Briefly, the serum samples were first thawed at room temperature, and then, 5 *μ*L of the serum samples were used for enzymatic colorimetric assays and spectrophotometry. All reagents were provided by the manufacturer and were validated prior to analyses.

### 2.5. Gut Microbiome Analysis

Microbial genomic DNA was extracted from the feces samples (100 mg feces per subject) using QIAcube HT and QIAcube HT Purification Kits (QIAGEN, Germany), according to the manufacturer's instructions. The hypervariable regions V3–V4 of the bacterial 16S rRNA gene were amplified with the forward primer 338F (5′-ACTCCTACGGGAGGCAGCA-3′) and the reverse primer 806R (5′-GGACTAACHVGGGTWTCTAAT-3′) using the KAPA HiFi HS ReadyMix (Kapa Biosystems Ltd., London, UK). The amplification conditions were as follows: initial denaturation at 98°C for 2 min, followed by 25 cycles of denaturation at 98°C for 15 s, annealing at 55°C for 30 s, and extension at 72°C for 30 s, with a final extension at 72°C for 5 min. The amplicons were first verified by agarose gel electrophoresis using QIAxcel Advanced System and QIAxcel DNA Kits (QIAGEN, Germany) and then subjected to library preparation using the Nextera XT v.3 Library Preparation Kit (Illumina, San Diego, USA). Thereafter, the amplicon libraries were sequenced using the Illumina MiSeq platform (Illumina, San Diego, USA) according to the standard protocol of the manufacturer.

More than 20,000 clean reads were obtained for each amplicon library. The raw reads were demultiplexed, quality-filtered, and denoised into amplicon sequence variants (ASVs) using the Quantitative Insights Into Microbial Ecology version 2 (QIIME2) software. Alpha and beta diversity analyses, sequence alignment, and taxonomic classification were also used for analyzing the sequencing results. The QIIME2View program was used to perform principal component analysis (PCA) based on Bray–Curtis distances to estimate beta diversity, and the community composition at the phylum and family levels was visualized using bar graphs.

### 2.6. Statistical Analyses

Significant differences between datasets were analyzed using one-way ANOVA, and Tukey's test was applied for post hoc analysis. Differences were considered statistically significant at *P* < 0.05.

Spearman's correlation coefficient was determined for correlation analysis, and the correlation was considered statistically significant at *P* < 0.05. We used GraphPad Prism version 7 (La Jolla, United States) for the graphical representation of data and IBM SPSS Statistics for Windows version 25.0 for statistical analyses.

## 3. Results

### 3.1. Body Weight

**Figure 1:**
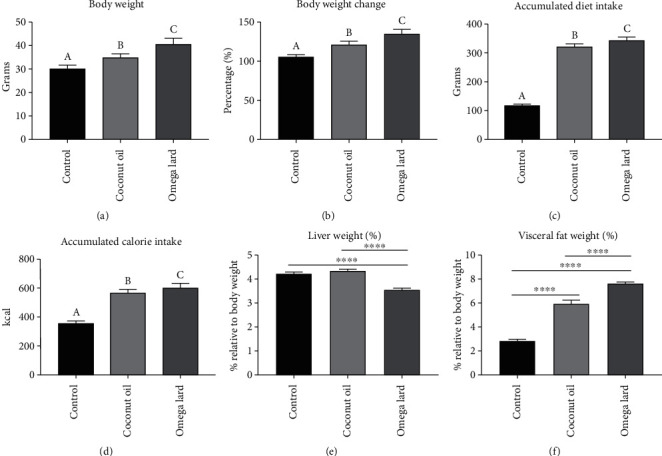
Differences in body weight, dietary intake, and organ weight. The differences in body weight (a), change in body weight (b), accumulated dietary intake(c), accumulated calorie intake (d), relative liver weight (e), and relative visceral fat weight (f) of all groups are shown. Control, coconut oil, and omega lard refer to the control, coconut oil-fed, and omega lard-fed groups, respectively. Body weight and change in body weight were estimated at the end of the experiment (four weeks). The accumulated dietary and calorie intakes were estimated daily. Liver and visceral fat tissues were harvested at the end of the experiment, and their weights were measured relative to the body weight of the mice. The number of mice was 8, 7, and 8 in the control, coconut oil-fed, and omega lard-fed groups, respectively. Data are presented as mean ± SEM. Uppercase letters and asterisk indicate significant differences between groups (*P* < 0.05).

The mice in the omega lard-fed group exhibited the maximum increase in body weight (35%), followed by those fed on coconut oil (approximately 22%) and the control group (approximately 6%) (Figures 1(a) and 1(b)) (Supplementary Table S1 and S2).

### 3.2. Diet and Calorie Intake

The omega lard-fed group exhibited the highest accumulated dietary intake and accumulated calorie intake throughout the experiment, followed by the coconut oil-fed and control groups (Figures 1(c) and 1(d)) (Supplementary Table S3 and S4).

As shown in Table 1, the coconut oil and omega lard diets provided lesser calories than the chow diet fed to mice in the control group, which resulted in higher dietary intake in mice fed with coconut oil and omega lard diets, to compensate for the fewer calories supplied by these dietary components.

### 3.3. Liver and Visceral Fat Weight

The percentage of liver weight to the body weight of the mice in the omega lard-fed group was significantly lower compared to those in the control and coconut oil-fed groups (*P* < 0.05) (Figure 1(e)). In contrast, the relative weight of visceral fat of mice in the omega lard-fed group was significantly higher than the control and coconut oil-fed groups (*P* < 0.05) (Figure 1(f)) (Supplementary Table S6).

### 3.4. Glucose and Serum Lipid Profiles

**Figure 2:**
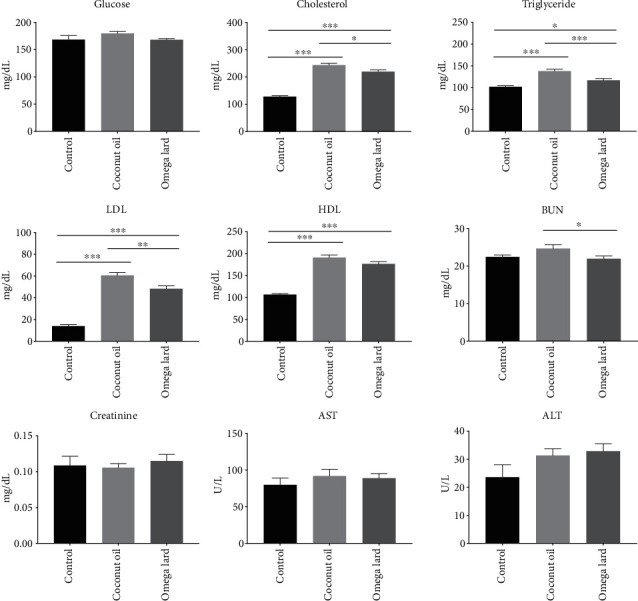
Differences in serum lipid profile and liver and kidney function enzymes. The differences in the levels of glucose, cholesterol, triglycerides, LDL, HDL, BUN, creatinine, ALT, and AST are shown. Control, coconut oil, and omega lard refer to the control, coconut oil-fed, and omega lard-fed groups, respectively. All parameters were analyzed using serum samples collected at the end of the experiment (four weeks). Data are presented as mean ± SEM. The number of mice was 8, 7, and 8 in the control, coconut oil-fed, and omega lard-fed groups, respectively. The serum level of each parameter is shown on the *y*-axis and the groups are indicated on the *x*-axis. Asterisks indicate significant differences between groups (*P* < 0.05).

There was no significant difference in serum glucose levels between the groups. However, the highest serum cholesterol, triglyceride, LDL, and HDL levels were noted in the coconut oil-fed group, and these levels were significantly higher than those of the mice in the control and omega lard-fed groups (*P* < 0.05) (Figure 2). In addition to the serum lipid profile, the omega lard-fed group exhibited a significantly lower BUN level compared to the coconut oil-fed group (*P* < 0.05) (Figure 2). Nonetheless, creatinine levels and the activities of ALT and AST exhibited no significant difference between the groups (Supplementary Table S5).

### 3.5. Analysis of Hepatic Macrovesicular Steatosis

**Figure 3:**
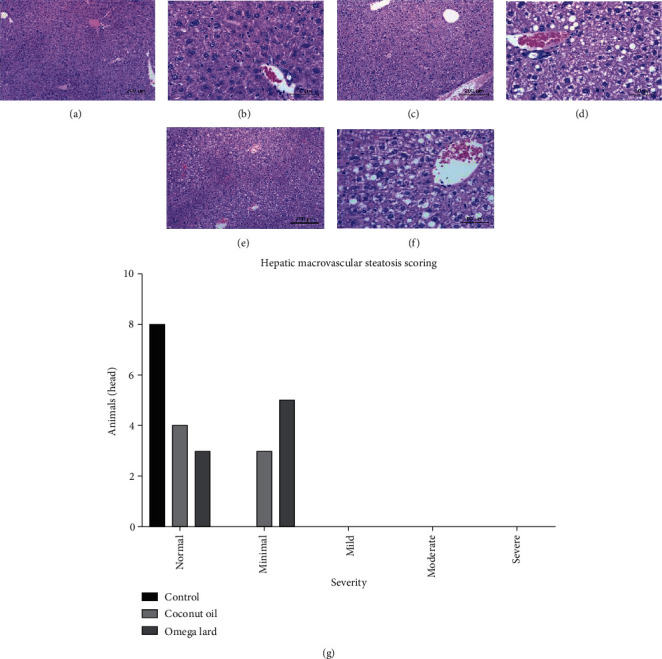
Hepatic macrovesicular steatosis. The histopathological staining of liver tissues showing hepatic macrovesicular steatosis in the control group at 10x (a) and 40x (b), coconut oil-fed group at 10x (c) and 40x (d), omega lard-fed group at 10x (e) and 40x (f) and the hepatic macrovesicular steatosis scores (g). Increased incidences of hepatic macrovesicular steatosis were observed in both the coconut oil- and omega lard-fed groups compared to the control group. The hepatic macrovesicular steatosis scores were consistent, showing a slightly increased incidence of steatosis in both the coconut oil- and omega lard-fed groups. No significant difference between the coconut oil- and omega lard-fed groups was observed (chi-square test, *P* < 0.05). The number of mice is presented on the *y*-axis and the severity of hepatic macrovesicular steatosis on the *x*-axis. Liver tissues were harvested from all animals at the end of the experiment (four weeks). The number of animals was 8, 7, and 8 in the control, coconut oil-fed, and omega lard-fed groups, respectively.

The results of the analysis of hepatic macrovesicular steatosis were normal in all groups, and only minimal changes in hepatic macrovesicular steatosis were observed in mice of the coconut oil- and omega lard-fed groups. However, the hepatic macrovesicular steatosis scores showed no significant difference between the coconut oil- and omega lard-fed groups (Figure 3) (Supplementary Table S7).

### 3.6. Fecal Microbiome Profile

**Figure 4:**
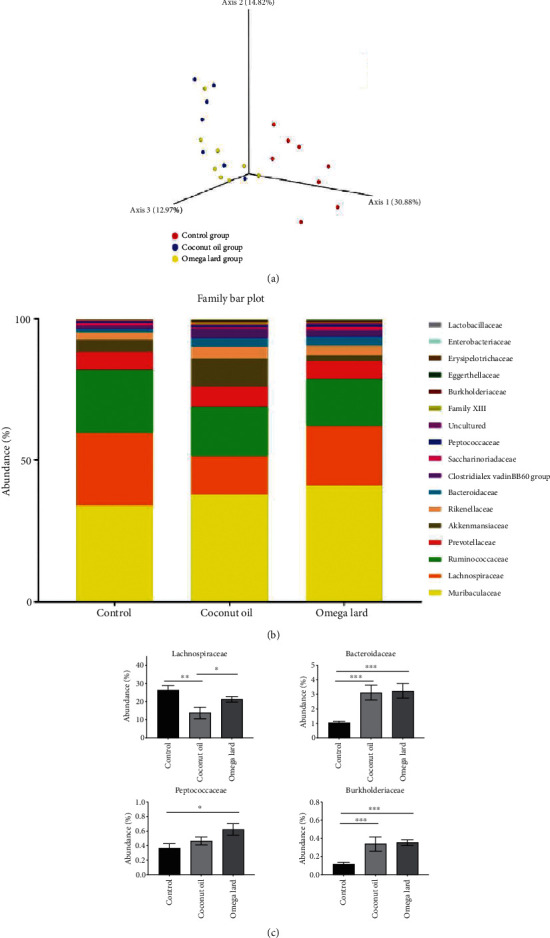
Microbiota analysis. Principal component analysis (PCA) of the fecal microbiome (a) is shown. Control, coconut oil, and omega lard represent the control, coconut oil-fed, and omega lard-fed groups, respectively. All feces samples were collected at the end of the experiment (four weeks). The number of animals was 8, 7, and 8 in the control, coconut oil-fed, and omega lard-fed groups, respectively. The PCA of the fecal microbiome was performed using Bray–Curtis measurements. The control, coconut oil-fed, and omega lard-fed groups are represented by red, blue, and yellow circles, respectively. The relative abundance of bacterial families in the fecal microbiome (b) is shown, where each bacterial family is indicated by a different color. The comparison of the relative abundance of bacterial families in the fecal microbiome between the groups is shown for *Lachnospiraceae*, *Bacteroidaceae*, *Peptococcaceae*, and *Burkholderiaceae* (c). The abundance (%) of the bacterial population is indicated on the *y*-axis and the groups on the *x*-axis. Data are presented as mean ± SEM. Asterisks indicate significant differences between groups (*P* < 0.05).

A 3D PCA revealed differences in fecal microbiomes of the control and experimental groups (Figure 4(a)). The fecal microbiome of mice belonging to the control group was separated from those of mice belonging to the coconut oil- and omega lard-fed groups, which overlapped.

### 3.7. Bacterial Abundance in the Microbiome

The fecal microbiome of the control group exhibited the maximum abundance of *Muribaculaceae* (37.64%), followed by *Lachnospiraceae* (20.30%), *Ruminococcaceae* (18.59%), *Prevotellaceae* (6.74%), *Akkermansiaceae* (5.42%), *Rikenellaceae* (3.35%), *Bacteroidaceae* (2.47%), Clostridiales vadin BB60 (2.17%), *Saccharimonadacea*e (1.00%), *Peptococcaceae* (0.49%), and *Burkholderiaceae* (0.27%) (Figure 4(b)) (Supplementary Table S8).

In contrast to the control group, the relative abundance of *Lachnospiraceae* was significantly reduced in the coconut oil-fed group, whereas the relative abundance of *Bacteroidaceae* and *Burkholderiaceae* was significantly increased (Figure 4(c)). The omega lard-fed group exhibited a significant increase in the relative abundance of *Bacteroidaceae*, *Peptococcaceae*, and *Burkholderiaceae* compared to the control group (Figure 4(c)). Additionally, the omega lard-fed group exhibited a significant increase in the relative abundance of *Lachnospiraceae* compared to the coconut oil-fed group (*P* < 0.05) (Figure 4(c)).

### 3.8. Correlation between Serum Lipid Profile and Fecal Microbiome

**Figure 5:**
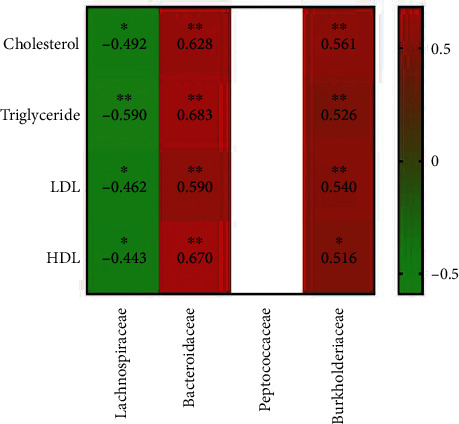
Correlation between microbiome and serum lipid content. Spearman's correlations between fecal microbiome and levels of different serum lipids are shown. ^∗^ indicates *P* < 0.05 and ^∗∗^ indicates *P* < 0.01.

Spearman's correlation analysis indicated significant positive correlations between the relative abundance of *Bacteroidaceae* and *Burkholderiaceae* and serum lipid content (*P* < 0.05) (Figure 5). However, significant negative correlations were observed between the relative abundance of *Lachnospiraceae* and the levels of cholesterol, triglycerides, LDL, and HDL (*P* < 0.05) (Figure 5) (Supplementary Table S9).

## 4. Discussion

This study indicated that the levels of serum cholesterol, triglycerides, and LDL were significantly reduced (*P* < 0.05) in the omega lard-fed group compared to the coconut oil-fed group. Moreover, in this study, increasing dietary fat levels improved serum lipid profile, especially in the coconut oil-fed group. This finding was similar to those of other studies, which indicated elevated levels of serum cholesterol two weeks after starting an HFD regimen [17, 18]. Although the elevated cholesterol levels in this study were not similar to the standard levels reported in hypercholesterolemia, we observed a significant increase that was sufficient for the comparison between the coconut oil- and omega lard-fed groups.

Omega-3 fatty acids might play a role in altering the levels of serum lipids, especially ALA, which is contained in the omega-3-rich pork lard [19]. However, the triglyceride levels of the omega lard-fed group were similar to those of the control group. The ALA in omega-3-rich pork lard, when used as a dietary ingredient, might reduce the levels of serum lipid at a higher magnitude compared with the ALA in coconut oil [20, 21]. Similar results were reported in several preclinical trials. For example, supplemented flaxseed oil, which is rich in ALA, reduced cholesterol and triglyceride levels in mice after feeding them an HFD for 16 and 18 weeks [22, 23]. Feeding an HFD supplemented with flaxseed meals, containing a high amount of ALA, for 14 and 24 weeks also reduced cholesterol and triglyceride levels in *LDLr*-/- mice [24]. Other preclinical trials on rabbits also reported similar results upon dietary supplementation with flaxseed meals for 6, 8, and 16 weeks [25].

Several studies have elucidated the mechanisms underlying the reduction in serum lipid content by ALA. One of the well-known mechanisms indicated that the reduction in serum cholesterol and triglyceride levels resulted from the suppression of sterol regulatory element binding protein (SREBP) transcriptional factors, which regulate several genes involved in cholesterol and triglyceride biosyntheses and uptake [26, 27]. According to this study, the consumption of ALA—an SREBP inhibitor—inhibited cholesterol and triglyceride biosyntheses and uptake, which, in turn, reduced serum cholesterol and triglyceride levels.

We did not observe any effect of HFD on liver function, as indicated by no significant difference in ALT and AST levels between the groups. Similarly, no effect of HFD was observed on kidney function; this was indicated by no significant difference in the levels of BUN and creatinine between the groups. As reported previously, high consumption of omega-3 fatty acids for 24 weeks, especially EPA and DHA, did not reveal any liver or kidney damage in a clinical setting [28]. A study on chronic high-fat feeding in rats, using 60% fat-based HFD for six weeks, revealed no significant change in renal function and early stages of renal diseases [29].

However, the weight of the liver relative to that of the body was significantly reduced in the omega lard-fed group compared to the control and coconut oil-fed groups (*P* < 0.05). This finding was similar to those of the studies on the effects of HFD+garlic oil [30], HFD+ginger oil [31], and HFD+microalgal oil [32] on protection against NAFLD. In contrast to the liver, the omega lard-fed group exhibited significantly higher visceral fat weight relative to the body weight, demonstrating that excessive fat was not stored in the liver but in the form of visceral fat.

The hepatic macrovesicular steatosis scores of coconut oil- and omega lard-fed groups were not significantly different. This implied that the scores resulted from high dietary fat content in the diet and not the presence of omega-3-rich pork lard. Combining the results of the relative weights of liver and visceral fat with the insignificant differences in hepatic macrovesicular steatosis scores between both the HFD groups, we suggest that omega-3-rich pork lard can serve as an alternative to coconut oil for NAFLD-concerned consumers.

The results of microbiome analysis were similar to those of previous studies. Our results indicated that the majority of the microbiome was composed of the families *Muribaculaceae*, *Lachnospiraceae*, *Prevotellaceae*, and *Ruminococcaceae*; this composition was similar to the microbiome composition of mice fed with a low-fat diet [33]. In this study, a significant increase in the abundance of *Peptococcaceae* was observed in the omega lard-fed group compared to the control group. This was similar to a study on obese mice fed with an HFD for 12 weeks [34]. Moreover, we also observed an increase in the abundance of *Bacteroidaceae*, which was similar to a study on rats fed with an HFD for 16 weeks [35].

Nonetheless, the abundance of *Akkermansiaceae* in the HFD groups was reduced in this study. In mice with the lean phenotype, the reported abundance of *Akkermansiaceae* was high [36]. Another study also revealed a similar finding, wherein the abundance of *Akkermansiaceae* decreased in the HFD group [37]. Although this study noted an increase in the abundance of *Akkermansiaceae* in the coconut oil-fed group, an HFD-fed group, their abundance was reduced to a similar level as the control and omega lard-fed groups. Previous studies suggested that the abundance of *Akkermansia* in the gut microbiome of both humans and rodents increased with the consumption of EPA and DHA, but not ALA [38]. Thus, the absence of EPA and DHA in the omega-3-rich pork lard used in this study might explain the similarity in the abundance of *Akkermansia* in the control and omega lard-fed groups. We hypothesized that using pigs, from which omega-3-rich pork lard was obtained, fed with tuna oil- or microalgae-fortified feed, both of which contain high levels of EPA and DHA, would have increased the abundance of *Akkermansia* in the omega lard-fed groups.

The increase in the abundance of *Bacteroidaceae* in coconut oil- and omega lard-fed groups was similar to another study [39]. Moreover, the positive correlations between the abundance of *Bacteroidaceae* and serum cholesterol, triglyceride, LDL, and HDL levels were reported in other preclinical trials involving rats and hamsters [40]. The findings of this study also agree with those of a clinical trial, wherein an increased abundance of *Bacteroidaceae* and increased cholesterol levels were observed in patients with hyperlipidemia [41]. This illustrates a positive correlation between the abundance of *Bacteroidaceae* and hyperlipidemia.

In addition to HFD, high dietary fat and animal protein are the determinants of the abundance of *Bacteroidaceae*. A preclinical study using a mice model reported an association between high-fat, high animal-based protein, and an increase in the abundance of *Bacteroidaceae* [42]. Similar findings were also reported in clinical settings where long- or short-term high animal fat diets were associated with an increased *Bacteroidaceae* population [43, 44]. Thus, our findings can be attributed to high dietary fat and animal protein content, which favored the increase in the abundance of *Bacteroidaceae* and serum lipid content.

In this study, the HFD regimen altered the composition of the microbiome, which was evident from the increased abundance of *Bacteroidaceae* (Figure 4(c)). This finding was similar to those of other studies, wherein increasing dietary fat increased the abundance of *Bacteroidaceae* because increasing dietary fat increased bile acid release for the emulsification and digestion of lipids [45]. Although bile acid exhibits antimicrobial activity [46], the abundance of *Bacteroidaceae* increased in the microbiome of HFD-fed mice because *Bacteroidaceae* are tolerant to bile acid.

In contrast to *Bacteroidaceae*, the abundance of *Lachnospiraceae* reported in this study exhibited an opposite trend; compared to the control group, a significant decrease in the abundance of *Lachnospiraceae* was observed in the coconut oil-fed group. Other studies have also reported a decrease in the abundance of *Lachnospiraceae* and an increase in cholesterol levels in C57BL/6 mice fed with an HFD [47]. However, a preclinical study showed that upon switching from an HFD to a balanced chow diet, the abundance of *Lachnospiraceae* was restored [48]. Another preclinical study that determined the effects of nutrients on *Lachnospiraceae* reported an increase in its abundance in C57BL6 mice fed with an HFD supplemented with a lipase inhibitor [49]. According to the findings of [49], the abundance of *Lachnospiraceae* in the microbiome was diet-dependent. Nevertheless, in the present study, compared to the control group, the omega lard-fed group exhibited an insignificant decrease in the abundance of *Lachnospiraceae* although the mice were fed with an HFD. This elucidated the effects of ALA as a component of the omega lard diet. In addition to the reduction in the levels of cholesterol, triglycerides, and LDL, ALA might be able to reverse the abundance of *Lachnospiraceae* caused by HFD.

The composition of the gut microbiome can illustrate various conditions of the host, particularly inflammation and dysbiosis, which are represented by an increased abundance of *Bacteroidetes* and *Proteobacteria* and an altered abundance of *Firmicutes* [50]. In contrast, an increased abundance of *Lachnospiraceae* indicated increased production of SCFAs [38, 51], especially butyrate, which has been reported to be involved as an anti-inflammatory agent in the gut [52] during inflammatory bowel disease (IBD) [53]. According to the gut microbiome analysis in this study, omega-3-rich pork lard exhibited the ability to restore the abundance of *Lachnospiraceae* that resulted from feeding on an HFD, which can be translated to a high SCFA production and protection against disorders involving gut inflammation, including IBD. Thus, the findings of this study can provide insights for further research on the association between the omega-3-rich pork lard, the abundance of *Lachnospiraceae*, and the amelioration of IBD.

Several reports have found that a combination of DHA and EPA significantly ameliorated NAFLD abnormalities in clinical settings for both children [54, 55] and adult patients [56]. ALA also exhibits similar NAFLD-protective properties as reported in a clinical trial with chia supplementation [57] and other preclinical trials with perilla oil- [58] and linseed oil-supplemented diets [59]. Based on our findings of improved levels of serum cholesterol, triglycerides, and LDL; increased relative liver weight; and high hepatic macrovesicular steatosis scores in the mice model, we suggest replacing the oils used in the preparation of the Western diet with omega-3-rich pork lard to increase ALA consumption, which will provide several health benefits to the consumers.

## 5. Conclusions

The present study revealed that including omega-3 fatty acids in the diet, not in the form of supplementation but through consumption of omega-3-rich pork lard, lowers serum lipid levels, including the levels of cholesterol, triglycerides, and LDL. Furthermore, this change in diet does not damage liver and kidney functions. However, a diet rich in omega-3 fatty acids significantly decreases liver weight relative to body weight, suggesting less fat accumulation in the liver. Moreover, omega-3 fatty acids also alter the composition of the gut microbiome by increasing the abundance of *Lachnospiraceae*, an important SCFA-producing bacterial taxon. Overall, these findings will contribute to improving the understanding of the effects of omega-3-enriched food ingredients, for example, pork lard, which can also exhibit beneficial effects in a preclinical setting.

## Figures and Tables

**Table 1:** Fatty acid composition of the oils used in this study.

Fatty acids (mg/100 g of oil)	Palm oil	Coconut oil	Omega lard
C4:0 butyric acid	0.000	0.000	0.000
C6:0 caproic acid	0.000	0.774	0.000
C8:0 caprylic acid	0.028	7.840	0.015
C10:0 capric acid	0.027	5.790	0.111
C12:0 lauric acid	0.244	46.700	0.425
C14:0 myristic acid	0.891	19.000	2.190
C16:0 palmitic acid	36.000	9.630	27.400
C16:1 palmitoleic acid	0.195	0.000	1.330
C18:0 stearic acid	3.990	2.750	16.700
C18:1 oleic acid	41.800	6.530	31.800
C18:2 linoleic acid (omega 6)	14.700	1.840	14.000
C18:3 gamma-linolenic acid methyl ester (omega 6)	0.135	0.017	0.000
C18:3 alpha-linolenic acid (ALA) (omega 3)	0.625	0.000	2.090
C18:4 stearidonic acid (omega 3)	0.000	0.000	0.000
C20:0 arachidic acid	0.370	0.101	0.242
C20:1 eicosenoic acid	0.377	0.000	0.992
C20:2 eicosadienoic acid methyl ester (omega 6)	0.000	0.000	0.403
C20:3 eicosatrienoic acid (omega 3)	0.000	0.000	0.206
C20:4 eicosatetraenoic acid (omega 6)	0.000	0.000	0.225
C20:5 eicosapentaenoic acid (EPA) (omega 3)	0.000	0.000	0.136
C22:1 erucic acid	0.000	0.000	0.000
C22:3 docosatrienoic acid (omega 3)	0.000	0.000	0.000
C22:4 adrenic acid methyl ester (omega 6)	0.000	0.000	0.076
C22:5 docosapentaenoic acid (DPA) (omega 3)	0.000	0.000	0.276
C22:6 docosahexaenoic acid (DHA) (omega 3)	0.000	0.000	0.061
C24:1 nervonic acid	0.000	0.000	0.000

**Table 2:** Macronutrient energy distribution of diets used in this study.

Macronutrients	Control	High-fat diet with coconut oil	High-fat diet with omega lard
Protein (% kcal)	25.0	25.0	25.0
Fat (% kcal)	15.0	40.0	40.0
Carbohydrate (% kcal)	60.0	35.0	35.0
Calorie (kcal)	3,040.0	1,760.0	1,760.0

**Table 3:** Composition of semisolid coconut oil diet and omega lard diet.

Components	High-fat diet with coconut oil		High-fat diet with omega lard	
	Weight (g)	Energy (kcal)	Weight (g)	Energy (kcal)
Rice	448	620	448	620
Red bean	20	29	20	29
Carrot	11	4	11	4
Grain	20	29	20	29
Pea	11	8	11	8
Corn	11	18	11	18
Chicken breast	382	429	382	429
Coconut oil	99	889	0	0
Omega lard	0	0	99	889
Total	1,002	2,026	1,002	2,026

**Table 4:** The protein, carbohydrate, and fat content of ingredients used for coconut oil diet and omega lard diet with the total energy of 1,756 kcal/kg.

Components (100 g)	Energy (kcal/100 g)	Protein content (g)	Carbohydrate content (g)	Fat content (g)
Rice, steamed	138.41	2.24	31.75	0.27
Red bean, steamed	145.65	8.55	26.09	0.79
Carrot, steamed	37.98	1.64	7.34	0.23
Grain, boiled	148.99	4.59	27.30	2.39
Pea, boiled	78.96	5.22	13.62	0.40
Corn, boiled	166.80	3.30	35.70	1.20
Chicken breast, without skin, boiled	112.20	24.90	0.00	1.40
Coconut oil	900.00	0.00	0.00	100.00
Omega lard	900.00	0.00	0.00	100.00
Total (g)		50.44	141.80	106.68

## Data Availability

All supporting information has been provided as follows: Table S1: body weight; Table S2: body weight change (%); Table S3: accumulated diet intake; Table S4: accumulated calorie intake; Table S5: blood chemistry; Table S6: organ weight; Table S7: hepatic macrovesicular steatosis score; Table S8: abundance of bacterial taxa in the microbiome; Table S9: correlation between microbiome and serum lipid content; and Table S10: comparison between raw materials analyzed data and the USDA data.

## Abbreviations

CVD: Cardiovascular disease
NAFLD: Nonalcoholic fatty liver disease
HFD: High-fat diet
SCFA: Short-chain fatty acids
ALA: Alpha-linolenic acid
EPA: Eicosapentaenoic acid
DHA: Docosahexaenoic acid
LDL: Low-density lipoprotein cholesterol
HDL: High-density lipoprotein cholesterol
BUN: Blood urea nitrogen
ALT: Alanine aminotransferase
AST: Aspartate aminotransferase
ASV: Amplicon sequence variants
PCA: Principal component analysis
SREBP: Sterol regulatory element binding protein
IBD: Inflammatory bowel disease.

## Additional Points

*Highlights*. (1) Omega-3-rich pork lard altered the lipid profile of mice fed with a high-fat diet. (2) Omega-3-rich pork lard affected the gut microbiome. (3) Serum lipid content was positively correlated with the abundance of *Bacteroidaceae*. (4) Serum lipid content was negatively correlated with the abundance of *Lachnospiraceae*.

## Supplementary Materials

Supplementary Materials All supporting information has been provided as follows: Table S1: body weight; Table S2: body weight change (%); Table S3: accumulated diet intake; Table S4: accumulated calorie intake; Table S5: blood chemistry; Table S6: organ weight; Table S7: hepatic macrovesicular steatosis score; Table S8: abundance of bacterial taxa in the microbiome; Table S9: correlation between microbiome and serum lipid content; and Table S10: comparison between raw materials analyzed data and the USDA data.
