# Supplementary material for: Effects of Omega-3-Rich Pork Lard on Serum Lipid Profile and Gut Microbiome in C57BL/6NJ Mice
Source: Int J Food Sci. 2022 Nov 22;2022:9269968. doi: 10.1155/2022/9269968 (PMC9708346; doi:10.1155/2022/9269968)
Supplement: Supplementary Materials — All supporting information has been provided as follows: Table S1: body weight; Table S2: body weight change (%); Table S3: accumulated diet intake; Table S4: accumulated calorie intake; Table S5: blood chemistry; Table S6: organ weight; Table S7: hepatic macrovesicular steatosis score; Table S8: abundance of bacterial taxa in the microbiome; Table S9: correlation between microbiome and serum lipid content; and Table S10: comparison between raw materials analyzed data and the USDA data. [file 9269968.f1.zip › Table S10 - Comparison between raw material analyzed data and USDA data (1).pdf]

Supplementary table S10: Comparison between raw material analyzed data and USDA data

Components (100g)	Energy (kcal/100g)	Protein content (g)	Carbohydrate content (g)	Fat content (g)
Rice, steamed	138.41	2.24	31.75	0.27
USDA: Rice, white, cooked NDB#20445	130.00	2.69	28.20	0.28
Red bean, steamed	145.65	8.55	26.09	0.79
USDA: Beans, kidney, California red NDB#16033	127.00	8.67	22.80	0.50
Carrot, steamed	37.98	1.64	7.34	0.23
USDA: Carrots, cooked, boiled NDB#11125	35.00	0.76	8.22	0.18
Grain, boiled	148.99	4.59	27.30	2.39
USDA: Millet, cooked NDB#20032	119.00	3.51	23.70	1.00
Pea, boiled	78.96	5.22	13.62	0.40
USDA: Peas, green, cooked, boiled NDB#11811	84.00	5.36	15.60	0.22
Corn, boiled	166.80	3.30	35.70	1.20
USDA: Corn, sweet, white, cooked, boiled NDB#11902	97.00	3.34	21.70	1.41
Chicken breast, without skin, boiled	112.20	24.90	0.00	1.40
USDA: Chicken, breast, skinless, meat only, cooked NDB#5749	148.00	29.5	0.00	3.39
Coconut oil	900.00	0.00	0.00	100.00
USDA: Oil, coconut NDB#4047	892.00	0.00	0.00	99.10
Omega lard	900.00	0.00	0.00	100.00
USDA: Lard NDB#4002	902.00	0.00	0.00	100.00
