# Supplementary material for: Effects of Omega-3-Rich Pork Lard on Serum Lipid Profile and Gut Microbiome in C57BL/6NJ Mice
Source: Int J Food Sci. 2022 Nov 22;2022:9269968. doi: 10.1155/2022/9269968 (PMC9708346; doi:10.1155/2022/9269968)
Supplement: Supplementary Materials — All supporting information has been provided as follows: Table S1: body weight; Table S2: body weight change (%); Table S3: accumulated diet intake; Table S4: accumulated calorie intake; Table S5: blood chemistry; Table S6: organ weight; Table S7: hepatic macrovesicular steatosis score; Table S8: abundance of bacterial taxa in the microbiome; Table S9: correlation between microbiome and serum lipid content; and Table S10: comparison between raw materials analyzed data and the USDA data. [file 9269968.f1.zip › Table S2 - Body weight change (%).pdf]

Supplementary table S2: Body weight change (%)

Treatment	Mouse #	Day 0	Day 1	Day 2	Day 3
Control	1.00	100.00	99.65	97.22	100.35
	2.00	100.00	99.29	98.94	100.35
	3.00	100.00	101.75	103.50	106.29
	4.00	100.00	100.34	98.29	99.32
	5.00	100.00	102.42	102.77	101.04
	6.00	100.00	99.66	99.66	99.32
	7.00	100.00	99.30	100.70	101.74
	8.00	100.00	98.67	98.00	100.33
Coconut oil	1.00	100.00	96.62	96.24	94.36
	2.00	100.00	100.72	96.39	97.11
	3.00	100.00	93.42	93.09	93.09
	4.00	100.00	99.65	100.35	101.77
	5.00	100.00	100.34	100.67	101.01
	6.00	100.00	94.31	93.98	96.32
	7.00	100.00	99.65	98.60	101.40
Omega lard	1.00	100.00	98.58	98.22	103.20
	2.00	100.00	104.93	106.25	103.95
	3.00	100.00	102.81	103.16	102.81
	4.00	100.00	101.33	102.00	104.00
	5.00	100.00	98.38	96.44	99.68
	6.00	100.00	99.67	101.64	103.95
	7.00	100.00	101.63	104.90	105.23
	8.00	100.00	97.08	97.73	99.35

Unit = %

Day 4	Day 5	Day 6	Day 7	Day 8	Day 9
100.69	100.00	98.61	98.61	98.61	98.61
101.42	101.42	101.77	102.48	103.90	101.77
107.69	108.04	105.24	104.55	104.90	103.15
100.68	101.02	100.00	99.66	102.05	101.71
101.38	100.69	100.35	100.00	102.42	100.69
98.64	98.98	99.66	98.64	101.36	101.02
101.39	100.35	100.00	101.74	101.74	101.74
100.33	100.33	99.67	100.00	100.67	99.67
93.98	97.74	98.50	101.13	101.50	105.26
94.95	100.36	96.39	97.83	97.11	100.00
94.41	97.70	96.38	100.00	103.95	102.30
102.12	103.18	102.47	103.53	103.18	103.18
101.01	100.67	100.00	104.04	103.37	103.70
97.32	96.66	95.99	97.99	97.66	99.00
103.51	101.05	100.70	101.05	101.75	104.21
102.49	105.34	102.49	103.20	106.05	107.12
104.93	105.59	106.91	107.89	108.55	110.20
105.26	106.32	105.26	108.07	109.82	113.33
104.67	107.00	107.33	106.00	109.33	112.00
99.35	101.29	102.59	102.59	103.88	104.85
108.22	108.88	107.24	107.89	108.55	111.51
107.19	108.82	110.13	111.44	111.44	114.71
98.05	99.35	100.65	101.62	103.57	106.17

Day 10	Day 11	Day 12	Day 13	Day 14	Day 15
99.31	100.00	99.65	98.61	97.92	97.22
102.13	101.42	102.48	101.77	102.48	101.42
103.85	103.85	104.55	102.45	103.15	101.75
101.02	103.07	103.07	102.05	101.02	102.73
101.38	99.31	100.69	100.00	99.65	101.38
101.36	101.36	103.05	101.36	102.03	102.03
101.74	102.44	102.44	101.74	102.79	103.83
101.00	100.33	100.67	99.00	99.67	101.67
106.77	108.65	109.40	112.03	113.16	113.16
100.72	100.36	103.61	101.44	101.81	102.53
106.25	104.61	105.59	107.24	108.22	111.18
107.42	108.13	109.19	108.83	109.89	113.07
103.70	106.06	106.40	107.41	107.74	106.06
101.34	101.00	104.01	103.68	106.02	105.02
103.86	104.91	104.91	108.07	107.37	110.88
108.19	107.83	108.90	110.68	110.32	109.25
111.84	114.47	116.78	117.43	116.78	120.39
112.98	117.54	118.60	120.00	118.25	121.05
112.33	114.33	115.00	117.33	119.67	119.33
110.68	112.30	114.24	113.92	114.56	116.18
115.13	114.47	114.14	113.49	114.80	117.11
117.32	118.30	119.93	117.65	121.24	122.55
107.79	110.06	110.06	111.04	113.64	113.31

Day 16	Day 17	Day 18	Day 19	Day 20	Day 21
98.96	96.88	99.31	101.04	100.69	99.65
103.90	102.13	103.19	104.96	105.32	104.26
102.80	103.50	103.15	101.75	102.45	101.75
104.78	104.10	103.41	103.75	105.46	104.44
102.77	101.04	101.04	101.73	104.50	102.08
102.71	102.71	102.37	103.39	106.10	105.76
103.83	104.18	103.83	104.53	103.14	103.48
102.00	101.33	101.67	101.33	102.67	103.00
116.54	117.29	117.29	120.68	121.80	123.31
105.05	105.05	105.42	105.78	106.50	107.58
112.50	113.82	112.83	114.80	115.46	114.14
113.07	115.90	113.43	117.31	117.31	117.31
106.73	106.06	108.08	109.43	109.76	113.47
107.36	109.03	110.03	110.37	112.04	112.37
110.88	110.18	112.28	113.33	112.98	115.44
109.61	111.74	111.74	114.59	113.88	113.52
120.07	121.38	122.04	123.03	127.30	128.29
121.75	123.16	124.56	127.02	126.67	127.72
121.33	124.33	125.67	125.67	126.67	129.33
119.09	119.74	122.98	124.60	125.57	126.86
118.75	119.74	122.70	123.03	125.66	126.32
125.16	124.18	128.43	128.43	130.39	130.72
116.88	117.21	119.16	122.08	123.38	124.35

Day 22	Day 23	Day 24	Day 25	Day 26	Day 27
100.00	101.74	101.04	102.78	102.43	101.04
106.03	105.67	107.09	108.16	107.09	107.09
103.85	103.50	103.50	105.59	104.20	102.80
107.17	106.83	107.17	108.53	107.17	107.85
105.19	104.50	103.81	106.23	103.81	104.50
107.46	107.46	106.78	109.15	108.47	108.47
106.62	106.62	105.92	107.32	106.97	105.57
103.67	102.33	102.67	103.00	101.67	101.67
125.94	123.68	122.56	124.81	124.81	127.07
107.22	110.47	111.91	112.27	115.88	115.16
119.41	118.75	118.75	121.38	121.05	122.04
120.14	119.43	121.20	123.67	123.67	124.73
114.48	115.49	117.85	120.54	120.54	119.87
113.38	114.38	114.72	118.73	117.73	117.73
114.74	116.84	117.89	118.95	119.65	119.30
118.15	118.15	118.51	121.00	119.93	119.93
130.59	130.59	133.22	135.53	135.53	135.53
130.18	129.82	134.04	134.74	132.63	134.04
129.67	130.00	132.33	133.67	133.67	133.00
128.80	127.83	128.48	130.74	131.39	132.69
127.30	127.63	128.95	129.61	131.25	131.58
133.33	133.66	135.62	136.93	137.25	138.89
125.32	126.62	127.92	128.90	130.84	131.49

Day 28
102.43
110.28
104.55
109.22
105.88
108.81
105.57
102.33
130.08
117.33
120.07
125.44
118.86
120.74
122.11
123.84
139.80
138.25
138.00
135.60
133.88
142.16
133.12
