# Supplementary material for: Effects of Omega-3-Rich Pork Lard on Serum Lipid Profile and Gut Microbiome in C57BL/6NJ Mice
Source: Int J Food Sci. 2022 Nov 22;2022:9269968. doi: 10.1155/2022/9269968 (PMC9708346; doi:10.1155/2022/9269968)
Supplement: Supplementary Materials — All supporting information has been provided as follows: Table S1: body weight; Table S2: body weight change (%); Table S3: accumulated diet intake; Table S4: accumulated calorie intake; Table S5: blood chemistry; Table S6: organ weight; Table S7: hepatic macrovesicular steatosis score; Table S8: abundance of bacterial taxa in the microbiome; Table S9: correlation between microbiome and serum lipid content; and Table S10: comparison between raw materials analyzed data and the USDA data. [file 9269968.f1.zip › Table S4 - Accumulated calorie intake.pdf]

Supplementary table S4: Accumulated calorie intake

| Treatment | Mouse # | Day 1 | Day 2 | Day 3 | Day 4 |
|---|---|---|---|---|---|
| Control | 1.00 | 14.90 | 25.84 | 39.22 | 56.24 |
| | 2.00 | 13.07 | 26.45 | 39.82 | 54.11 |
| | 3.00 | 13.68 | 31.92 | 50.16 | 67.49 |
| | 4.00 | 14.90 | 27.06 | 41.04 | 55.63 |
| | 5.00 | 14.29 | 29.49 | 41.34 | 55.02 |
| | 6.00 | 13.68 | 27.06 | 39.82 | 51.07 |
| | 7.00 | 15.81 | 29.79 | 43.78 | 58.37 |
| | 8.00 | 12.16 | 25.84 | 18.54 | 31.62 |
| Coconut oil | 1.00 | 20.42 | 40.13 | 60.54 | 77.97 |
| | 2.00 | 23.58 | 42.42 | 66.35 | 86.94 |
| | 3.00 | 16.37 | 35.20 | 55.26 | 76.03 |
| | 4.00 | 23.06 | 45.58 | 64.24 | 84.48 |
| | 5.00 | 27.10 | 48.40 | 71.63 | 95.04 |
| | 6.00 | 20.77 | 42.24 | 65.12 | 86.24 |
| | 7.00 | 25.87 | 48.75 | 71.28 | 95.04 |
| Omega lard | 1.00 | 24.29 | 46.11 | 68.29 | 88.35 |
| | 2.00 | 15.84 | 41.01 | 62.30 | 80.78 |
| | 3.00 | 25.17 | 48.40 | 69.52 | 89.94 |
| | 4.00 | 28.86 | 55.97 | 64.42 | 89.41 |
| | 5.00 | 24.82 | 46.99 | 67.23 | 89.23 |
| | 6.00 | 24.46 | 49.81 | 75.15 | 100.67 |
| | 7.00 | 26.05 | 55.09 | 81.14 | 104.72 |
| | 8.00 | 18.13 | 25.87 | 48.75 | 68.11 |

Unit = kcal

| Day 5 | Day 6 | Day 7 | Day 8 | Day 9 | Day 10 |
|---|---|---|---|---|---|
| 69.31 | 79.04 | 89.68 | 102.45 | 114.91 | 127.07 |
| 68.40 | 81.47 | 93.33 | 109.44 | 120.69 | 133.15 |
| 82.99 | 92.42 | 101.84 | 114.30 | 125.86 | 137.10 |
| 68.10 | 79.95 | 90.90 | 103.36 | 115.52 | 127.68 |
| 67.18 | 78.43 | 89.07 | 103.36 | 114.61 | 126.77 |
| 63.54 | 76.61 | 88.46 | 102.75 | 115.22 | 127.98 |
| 71.74 | 84.21 | 97.28 | 110.66 | 124.03 | 137.10 |
| 45.90 | 57.46 | 72.35 | 85.73 | 97.89 | 110.96 |
| 99.62 | 119.68 | 140.10 | 161.22 | 184.27 | 202.75 |
| 108.59 | 126.19 | 144.14 | 163.33 | 182.34 | 199.94 |
| 96.80 | 115.46 | 136.05 | 160.34 | 176.88 | 198.53 |
| 105.78 | 123.38 | 142.74 | 160.51 | 178.99 | 200.64 |
| 114.93 | 134.29 | 157.17 | 177.58 | 197.47 | 217.89 |
| 106.13 | 125.66 | 145.90 | 165.26 | 183.92 | 203.10 |
| 114.05 | 131.47 | 151.71 | 171.07 | 189.55 | 207.33 |
| 108.77 | 125.31 | 143.26 | 163.68 | 181.28 | 199.58 |
| 99.09 | 121.26 | 140.62 | 160.51 | 182.16 | 205.74 |
| 111.58 | 132.88 | 153.82 | 176.00 | 198.53 | 218.77 |
| 112.11 | 134.29 | 154.53 | 177.06 | 198.53 | 218.94 |
| 112.46 | 135.70 | 157.52 | 179.34 | 200.99 | 225.10 |
| 124.08 | 145.20 | 164.91 | 186.74 | 210.14 | 233.73 |
| 127.42 | 144.14 | 164.56 | 184.80 | 207.50 | 230.03 |
| 89.41 | 111.23 | 130.77 | 152.42 | 175.47 | 197.82 |

| Day 11 | Day 12 | Day 13 | Day 14 | Day 15 | Day 16 |
|---|---|---|---|---|---|
| 140.14 | 152.30 | 162.34 | 173.28 | 184.53 | 196.69 |
| 145.01 | 157.78 | 168.11 | 181.18 | 193.65 | 207.02 |
| 149.26 | 162.64 | 175.41 | 187.87 | 200.03 | 215.23 |
| 141.36 | 153.52 | 165.38 | 175.10 | 189.39 | 203.38 |
| 137.71 | 149.26 | 161.42 | 173.58 | 188.18 | 203.07 |
| 141.06 | 153.52 | 165.98 | 178.75 | 191.82 | 205.20 |
| 149.57 | 162.03 | 174.50 | 185.74 | 199.42 | 212.80 |
| 123.42 | 135.28 | 147.14 | 159.60 | 174.50 | 187.57 |
| 222.29 | 242.70 | 263.65 | 284.24 | 305.36 | 326.66 |
| 217.18 | 235.49 | 255.73 | 274.38 | 294.80 | 316.62 |
| 217.01 | 236.02 | 256.43 | 276.14 | 299.90 | 323.31 |
| 219.47 | 237.60 | 256.78 | 276.50 | 301.31 | 322.08 |
| 237.60 | 258.02 | 280.72 | 301.66 | 321.90 | 343.55 |
| 222.29 | 243.41 | 263.65 | 283.71 | 306.06 | 330.00 |
| 226.16 | 248.34 | 265.41 | 289.52 | 313.81 | 336.69 |
| 217.01 | 240.06 | 257.84 | 274.91 | 293.22 | 312.22 |
| 225.81 | 241.30 | 263.82 | 287.06 | 312.22 | 333.34 |
| 240.94 | 261.01 | 282.48 | 301.14 | 321.90 | 343.90 |
| 240.59 | 260.83 | 282.66 | 304.66 | 326.83 | 348.83 |
| 241.82 | 264.18 | 285.30 | 307.12 | 329.12 | 352.53 |
| 255.90 | 277.20 | 299.20 | 322.26 | 343.55 | 367.49 |
| 250.98 | 273.86 | 295.33 | 319.09 | 341.62 | 364.14 |
| 220.70 | 242.70 | 264.35 | 286.00 | 308.88 | 332.82 |

| Day 17 | Day 18 | Day 19 | Day 20 | Day 21 | Day 22 |
|---|---|---|---|---|---|
| 208.54 | 221.31 | 234.69 | 248.06 | 259.31 | 272.08 |
| 219.18 | 232.26 | 246.54 | 259.01 | 270.26 | 283.63 |
| 229.82 | 242.59 | 252.62 | 264.78 | 275.42 | 287.58 |
| 215.84 | 228.00 | 239.86 | 252.02 | 263.26 | 277.55 |
| 214.93 | 227.70 | 240.46 | 252.62 | 264.78 | 278.16 |
| 217.97 | 231.04 | 244.11 | 258.70 | 271.47 | 285.76 |
| 226.18 | 237.73 | 251.10 | 262.35 | 275.73 | 290.02 |
| 200.03 | 213.10 | 225.26 | 236.82 | 251.41 | 265.09 |
| 349.89 | 368.54 | 390.02 | 411.31 | 431.02 | 451.97 |
| 336.51 | 353.41 | 370.48 | 389.66 | 406.56 | 426.27 |
| 344.96 | 362.03 | 379.63 | 398.64 | 413.78 | 433.84 |
| 343.38 | 358.34 | 382.27 | 399.87 | 416.42 | 437.01 |
| 362.03 | 380.51 | 402.51 | 424.51 | 443.87 | 466.75 |
| 352.18 | 365.90 | 387.20 | 408.50 | 427.15 | 448.45 |
| 356.40 | 375.76 | 396.35 | 415.54 | 435.60 | 456.19 |
| 331.76 | 351.12 | 372.06 | 390.90 | 409.90 | 429.44 |
| 353.23 | 374.70 | 398.82 | 420.64 | 442.99 | 463.23 |
| 362.91 | 385.09 | 407.09 | 428.91 | 448.80 | 467.63 |
| 369.25 | 390.02 | 410.43 | 429.97 | 451.44 | 469.04 |
| 371.36 | 394.42 | 416.94 | 438.94 | 460.59 | 482.06 |
| 394.94 | 416.94 | 439.30 | 460.77 | 482.24 | 503.18 |
| 384.38 | 406.91 | 427.15 | 449.33 | 469.39 | 489.28 |
| 354.46 | 376.46 | 401.28 | 423.98 | 447.04 | 468.69 |

| Day 23 | Day 24 | Day 25 | Day 26 | Day 27 | Day 28 |
|---|---|---|---|---|---|
| 286.37 | 299.14 | 312.21 | 324.67 | 336.22 | 348.08 |
| 295.79 | 308.26 | 322.24 | 335.31 | 347.17 | 360.54 |
| 300.35 | 311.30 | 326.50 | 339.87 | 349.90 | 361.76 |
| 291.23 | 302.18 | 307.95 | 319.50 | 332.27 | 345.04 |
| 291.54 | 303.09 | 317.38 | 328.02 | 340.18 | 351.12 |
| 299.44 | 312.51 | 328.02 | 342.00 | 356.29 | 369.06 |
| 303.39 | 316.77 | 330.14 | 343.82 | 354.16 | 366.02 |
| 277.25 | 288.50 | 301.26 | 313.12 | 323.76 | 335.01 |
| 468.86 | 486.29 | 504.59 | 523.78 | 544.02 | 566.19 |
| 445.28 | 464.99 | 484.88 | 506.70 | 526.59 | 547.18 |
| 452.85 | 469.39 | 490.16 | 508.82 | 527.82 | 545.07 |
| 455.84 | 475.20 | 494.56 | 514.10 | 533.98 | 555.28 |
| 488.75 | 511.46 | 531.52 | 552.46 | 574.82 | 592.77 |
| 467.98 | 488.05 | 512.16 | 533.81 | 555.10 | 579.22 |
| 475.38 | 495.97 | 516.74 | 536.98 | 556.34 | 577.81 |
| 448.10 | 467.28 | 488.93 | 508.99 | 529.06 | 551.76 |
| 485.58 | 507.94 | 530.99 | 552.99 | 574.99 | 601.39 |
| 487.70 | 511.63 | 533.46 | 554.40 | 575.17 | 600.34 |
| 490.51 | 510.75 | 535.04 | 558.45 | 577.81 | 601.92 |
| 503.01 | 523.60 | 546.66 | 569.36 | 589.25 | 610.37 |
| 523.78 | 544.54 | 566.72 | 589.60 | 613.54 | 636.06 |
| 510.22 | 530.82 | 554.75 | 574.99 | 598.22 | 617.23 |
| 491.74 | 513.74 | 537.33 | 560.38 | 586.61 | 607.38 |
