# Supplementary material for: Effects of Omega-3-Rich Pork Lard on Serum Lipid Profile and Gut Microbiome in C57BL/6NJ Mice
Source: Int J Food Sci. 2022 Nov 22;2022:9269968. doi: 10.1155/2022/9269968 (PMC9708346; doi:10.1155/2022/9269968)
Supplement: Supplementary Materials — All supporting information has been provided as follows: Table S1: body weight; Table S2: body weight change (%); Table S3: accumulated diet intake; Table S4: accumulated calorie intake; Table S5: blood chemistry; Table S6: organ weight; Table S7: hepatic macrovesicular steatosis score; Table S8: abundance of bacterial taxa in the microbiome; Table S9: correlation between microbiome and serum lipid content; and Table S10: comparison between raw materials analyzed data and the USDA data. [file 9269968.f1.zip › Table S5 - Blood chemistry.pdf]

Supplementary table S5: Blood chemistry

Treatment	Mouse #	Glucose	Cholesterol	Triglyceride	LDL (mg/dL)
Control	1.00	133.85	128.00	92.40	11.60
	2.00	170.20	140.90	106.30	19.20
	3.00	205.55	118.20	104.10	11.00
	4.00	172.30	136.20	107.50	21.00
	5.00	176.15	127.30	107.60	12.10
	6.00	149.90	135.60	104,1	15.70
	7.00	161.85	124.80	88.70	10.90
	8.00	177.45	114.30	105.20	10.50
Coconut oil	1.00	187.85	252.50	126.10	68.90
	2.00	179.90	241.50	152.90	55.70
	3.00	166.70	274.90	138.30	70.70
	4.00	180.55	244.50	137.70	61.80
	5.00	193.15	220.90	152.50	51.10
	6.00	180.40	234.80	135.90	60.10
	7.00	169.55	236.50	123.50	55.90
Omega lard	1.00	172.50	206.10	126.80	40.10
	2.00	172.75	233.90	122.60	56.80
	3.00	166.15	214.50	131.20	41.80
	4.00	173.20	209.50	114.80	38.90
	5.00	154.20	221.90	97.80	47.50
	6.00	168.10	237.50	122.60	51.90
	7.00	171.30	192.70	103.70	49.00
	8.00	166.40	243.10	115.10	59.90

HDL (mg/dL)	BUN (mg/dL)	reatinine (mg/dl	AST (U/L)	ALT (U/T)
109.00	22.60	0.08	73.50	17.50
116.80	23.40	0.18	133.70	54.20
99.10	19.30	0.10	83.10	20.00
107.40	22.60	0.12	95.20	21.70
105.40	21.90	0.08	53.60	15.60
111.70	23.10	0.08	59.40	20.10
106.50	22.80	0.09	81.90	20.80
95.50	23.80	0.14	58.80	18.90
209.90	24.30	0.09	91.20	40.40
198.80	29.50	0.11	135.00	33.30
209.10	21.20	0.09	77.40	34.70
179.90	25.00	0.12	113.80	34.70
167.80	23.60	0.12	72.40	24.60
184.50	22.90	0.09	84.80	29.70
188.10	26.30	0.12	68.10	22.00
170.90	26.40	0.15	113.70	23.40
184.30	21.40	0.12	72.40	36.10
173.90	20.10	0.13	105.90	35.70
174.40	21.20	0.08	103.00	32.60
180.40	23.60	0.10	76.80	28.60
194.00	21.30	0.12	96.40	45.30
143.50	20.30	0.08	70.00	37.40
190.20	21.30	0.14	73.80	24.10
