# Supplementary material for: Effects of Omega-3-Rich Pork Lard on Serum Lipid Profile and Gut Microbiome in C57BL/6NJ Mice
Source: Int J Food Sci. 2022 Nov 22;2022:9269968. doi: 10.1155/2022/9269968 (PMC9708346; doi:10.1155/2022/9269968)
Supplement: Supplementary Materials — All supporting information has been provided as follows: Table S1: body weight; Table S2: body weight change (%); Table S3: accumulated diet intake; Table S4: accumulated calorie intake; Table S5: blood chemistry; Table S6: organ weight; Table S7: hepatic macrovesicular steatosis score; Table S8: abundance of bacterial taxa in the microbiome; Table S9: correlation between microbiome and serum lipid content; and Table S10: comparison between raw materials analyzed data and the USDA data. [file 9269968.f1.zip › Table S6 - Organ weight.pdf]

Supplementary table S6: Organ weight

| Treatment | Mouse # | Liver weight | Visceral fat |
|---|---|---|---|
| Control | 1.00 | 4.17 | 2.55 |
| | 2.00 | 4.24 | 2.95 |
| | 3.00 | 4.11 | 3.30 |
| | 4.00 | 4.44 | 3.40 |
| | 5.00 | 4.32 | 2.63 |
| | 6.00 | 4.07 | 3.20 |
| | 7.00 | 4.32 | 2.34 |
| | 8.00 | 4.21 | 2.41 |
| Coconut oil | 1.00 | 4.35 | 6.51 |
| | 2.00 | 4.63 | 4.88 |
| | 3.00 | 4.17 | 7.87 |
| | 4.00 | 4.13 | 5.72 |
| | 5.00 | 4.42 | 5.18 |
| | 6.00 | 4.41 | 5.70 |
| | 7.00 | 4.17 | 5.26 |
| Omega lard | 1.00 | 3.41 | 6.60 |
| | 2.00 | 3.65 | 7.90 |
| | 3.00 | 3.42 | 7.38 |
| | 4.00 | 3.64 | 7.45 |
| | 5.00 | 3.57 | 7.72 |
| | 6.00 | 3.59 | 8.08 |
| | 7.00 | 3.61 | 8.09 |
| | 8.00 | 3.23 | 7.41 |

Unit = % relative to body weight
