# Supplementary material for: Effects of Omega-3-Rich Pork Lard on Serum Lipid Profile and Gut Microbiome in C57BL/6NJ Mice
Source: Int J Food Sci. 2022 Nov 22;2022:9269968. doi: 10.1155/2022/9269968 (PMC9708346; doi:10.1155/2022/9269968)
Supplement: Supplementary Materials — All supporting information has been provided as follows: Table S1: body weight; Table S2: body weight change (%); Table S3: accumulated diet intake; Table S4: accumulated calorie intake; Table S5: blood chemistry; Table S6: organ weight; Table S7: hepatic macrovesicular steatosis score; Table S8: abundance of bacterial taxa in the microbiome; Table S9: correlation between microbiome and serum lipid content; and Table S10: comparison between raw materials analyzed data and the USDA data. [file 9269968.f1.zip › Table S9 - Microbiome and lipid profile correlation.pdf]

Supplementary table S9: Microbiome and lipid profile correlation

| | Lachnospiraceae | | Bacteroidaceae | | Peptoco |
|---|---|---|---|---|---|
| | Coefficient | P-value | Coefficient | P-value | Coefficient |
| Cholesterol | -.492* | 0.017 | .628** | 0.001 | 0.309 |
| Triglyceride | -.590** | 0.003 | .683** | 0.000 | 0.087 |
| LDL (mg/dL) | -.462* | 0.026 | .590** | 0.003 | 0.343 |
| HDL (mg/dL) | -.443* | 0.034 | .670** | 0.000 | 0.392 |

| ccaceae | Burkholderiaceae | |
|---|---|---|
| P-value | Coefficient | P-value |
| 0.151 | .561** | 0.005 |
| 0.695 | .526** | 0.010 |
| 0.109 | .540** | 0.008 |
| 0.064 | .516* | 0.012 |
